# Supplementary figures and images for: EGCG’s anticancer potential unveiled: triggering apoptosis in lung cancer cell lines through in vitro investigation
Source: PeerJ. 2025 Mar 26;13:e19135. doi: 10.7717/peerj.19135 (PMC11954466; doi:10.7717/peerj.19135)

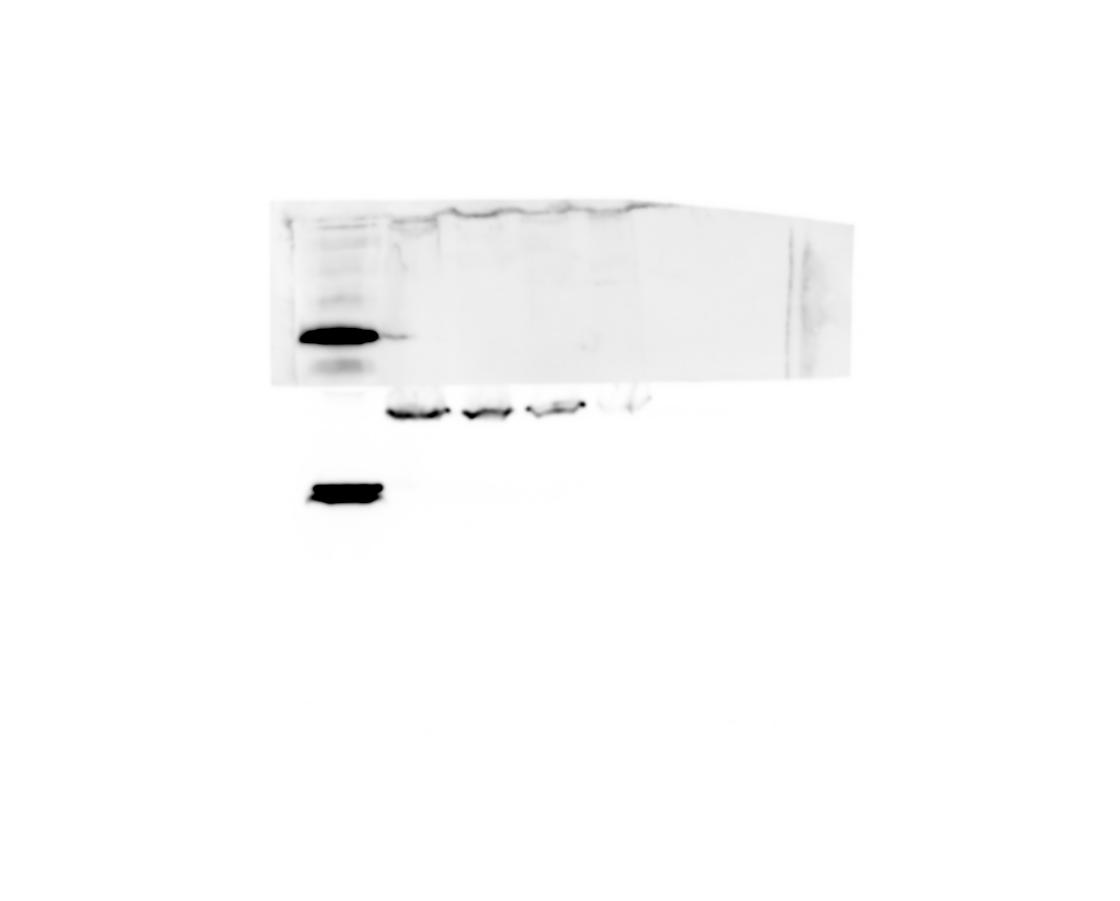

Supplement: Supplemental Information 2 [file peerj-13-19135-s002.zip › IMG-20240427-WA0028.jpg]

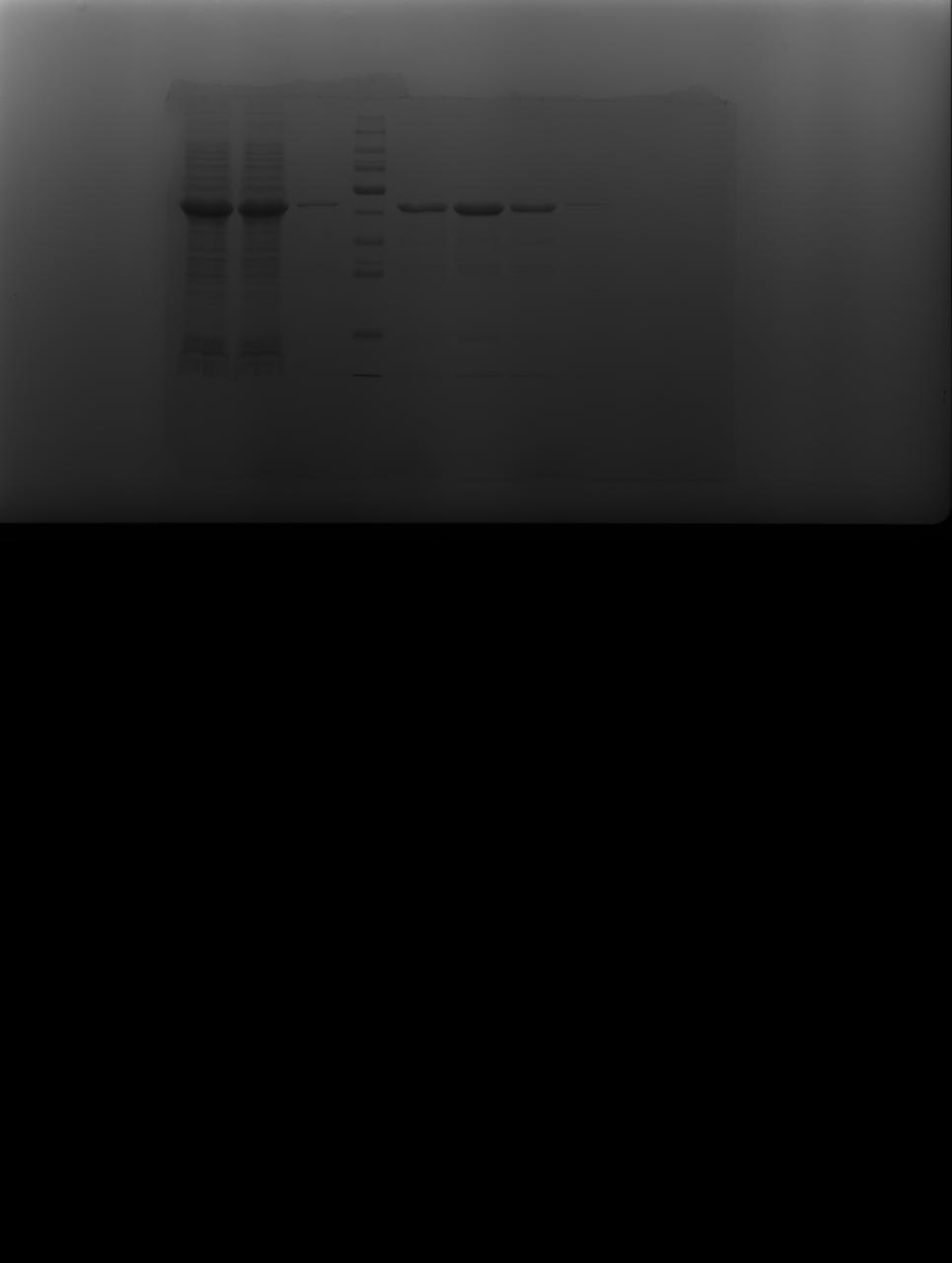

Supplement: Supplemental Information 2 [file peerj-13-19135-s002.zip › IMG-20240427-WA0029.jpg]

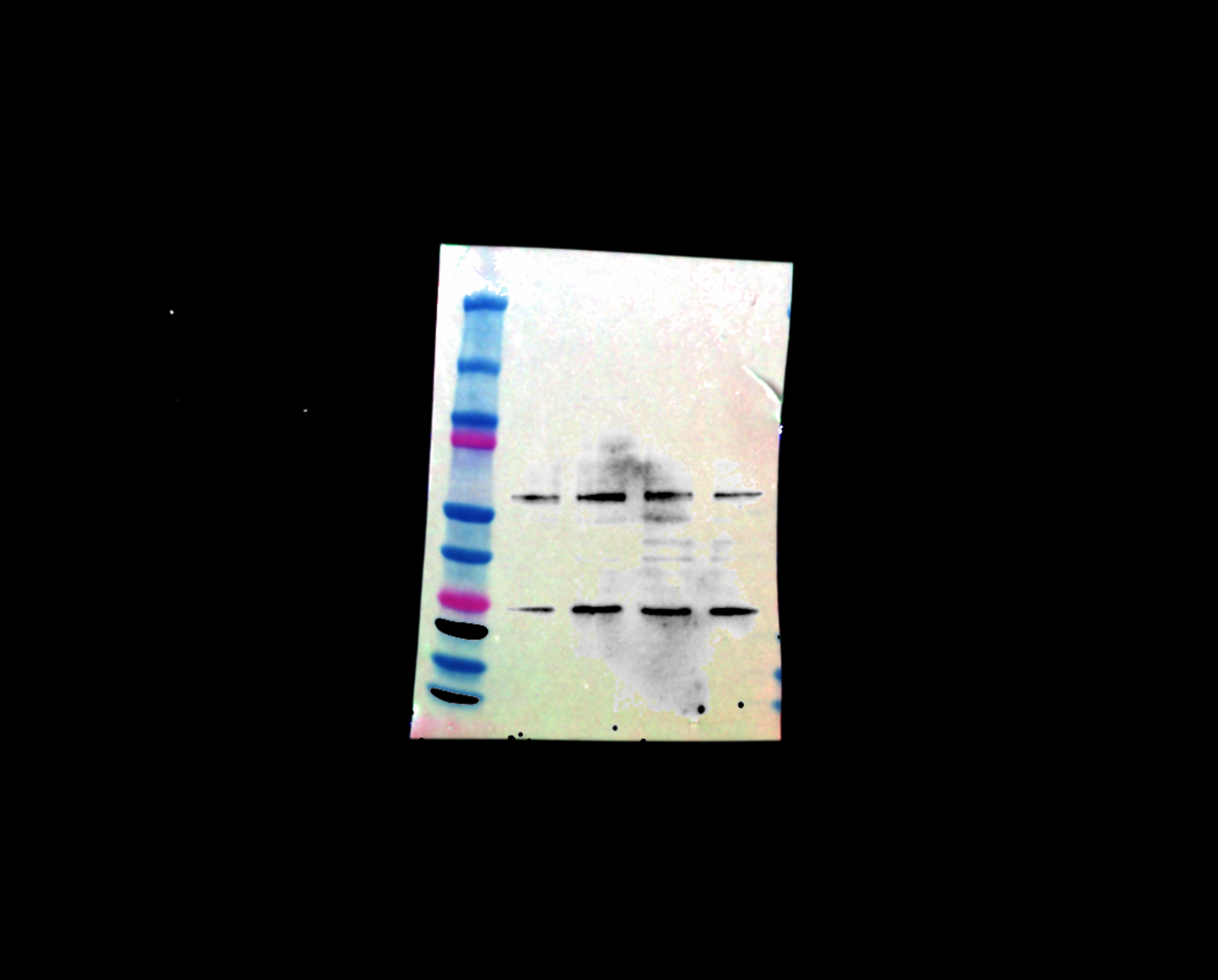

Supplement: Supplemental Information 2 [file peerj-13-19135-s002.zip › IMG-20240427-WA0030.jpg]

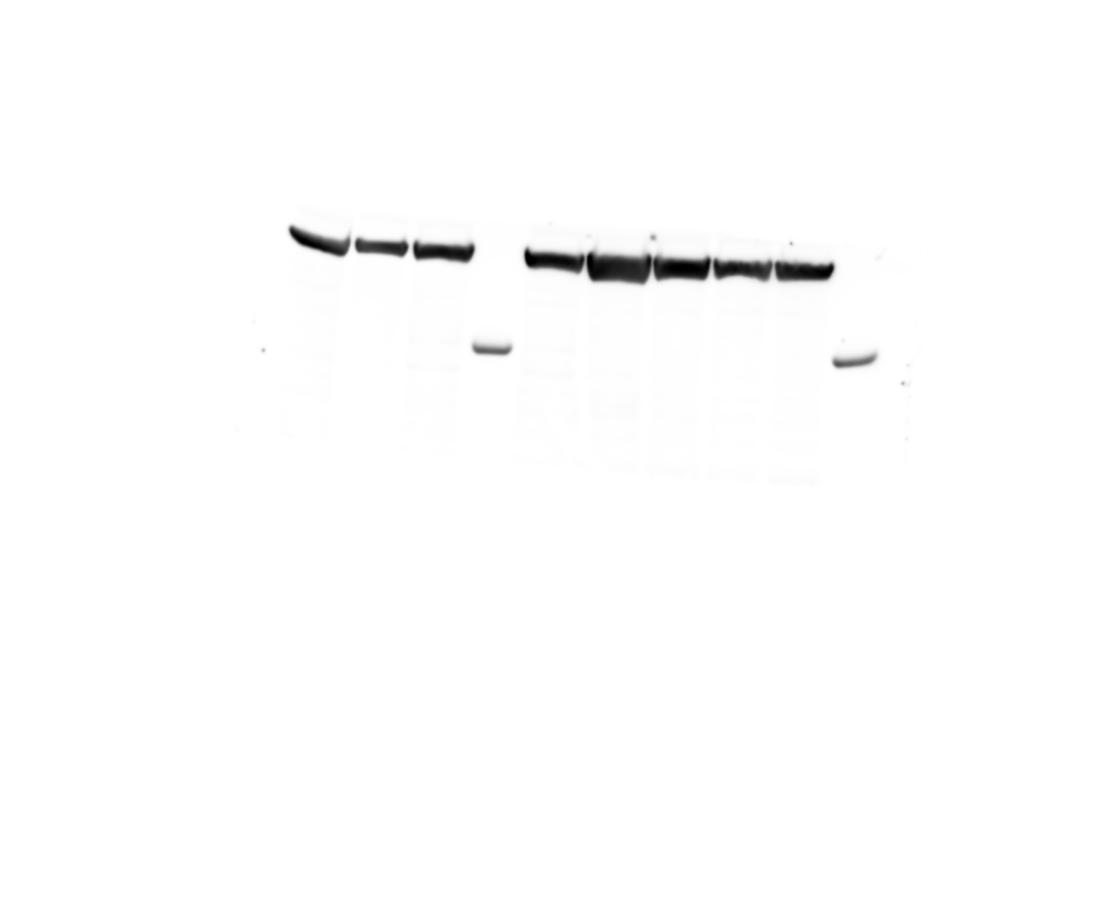

Supplement: Supplemental Information 2 [file peerj-13-19135-s002.zip › IMG-20240427-WA0031.jpg]

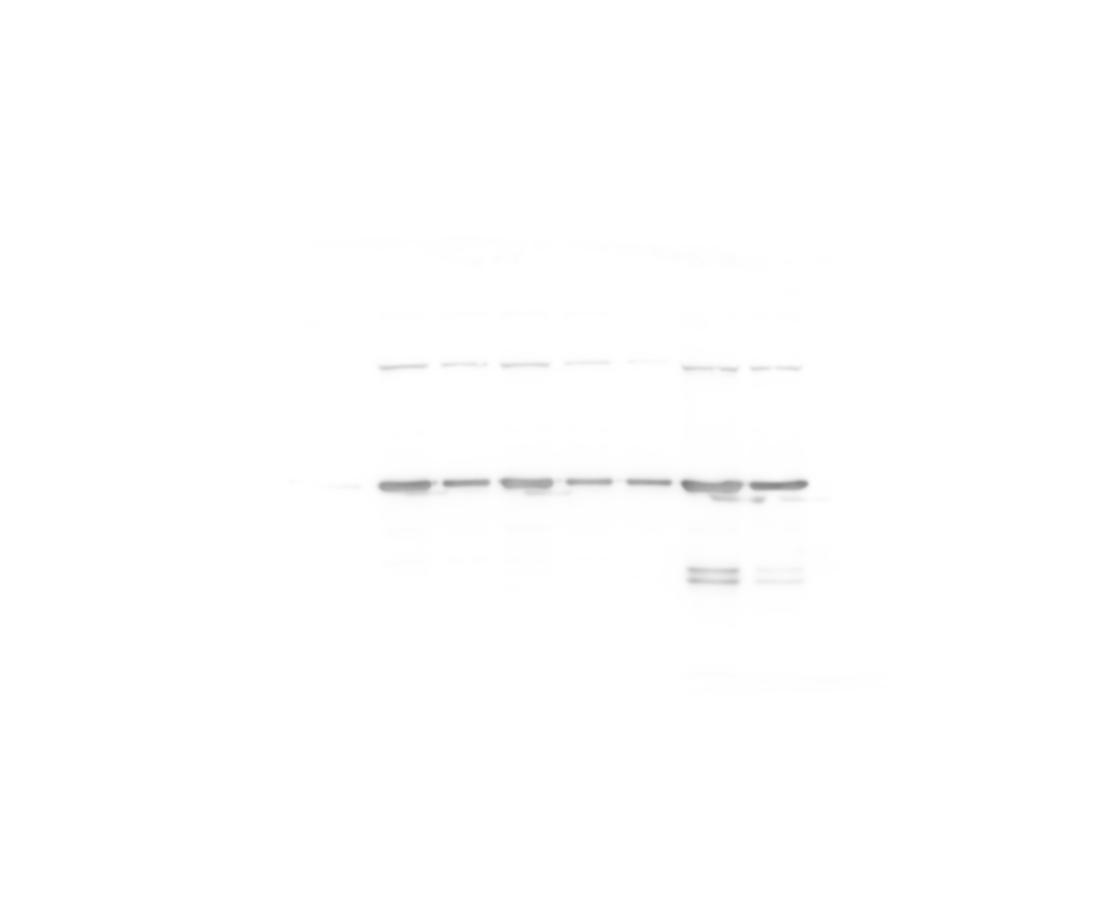

Supplement: Supplemental Information 2 [file peerj-13-19135-s002.zip › IMG-20240427-WA0032.jpg]

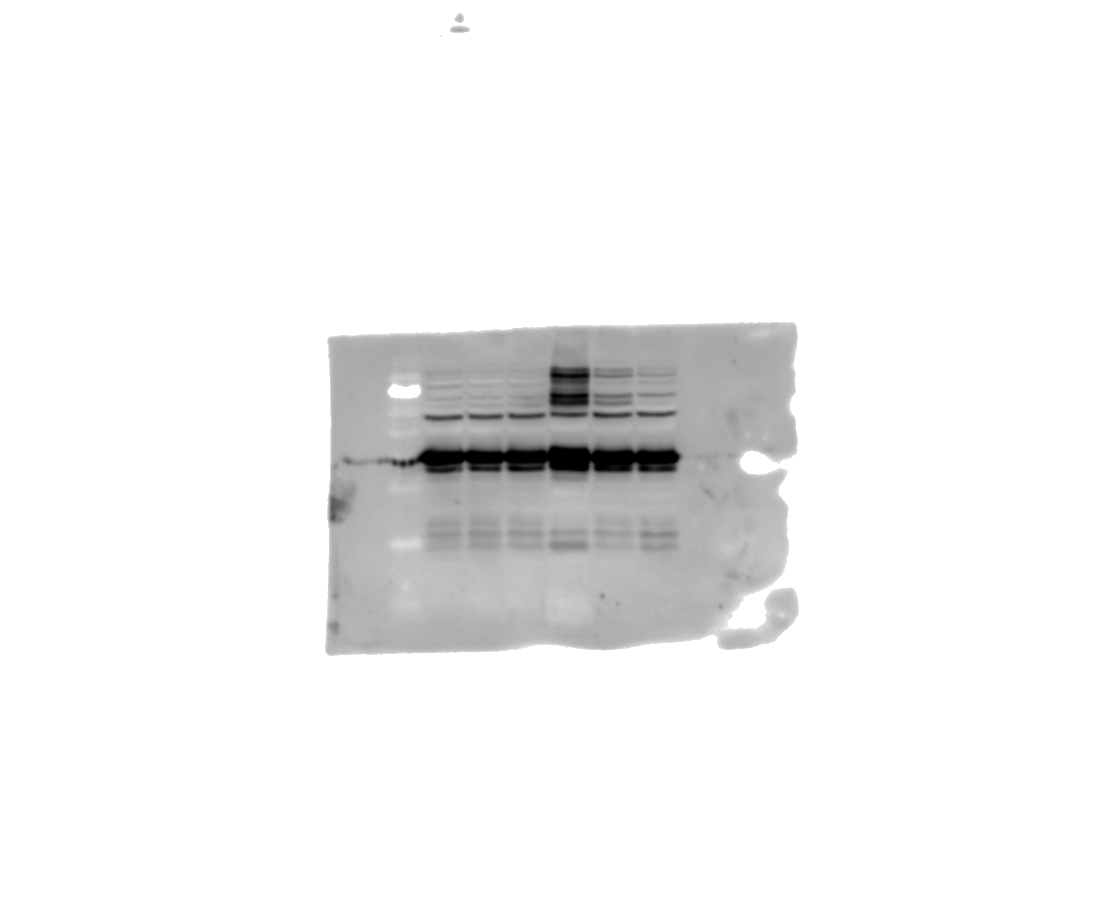

Supplement: Supplemental Information 2 [file peerj-13-19135-s002.zip › IMG-20240427-WA0033.jpg]

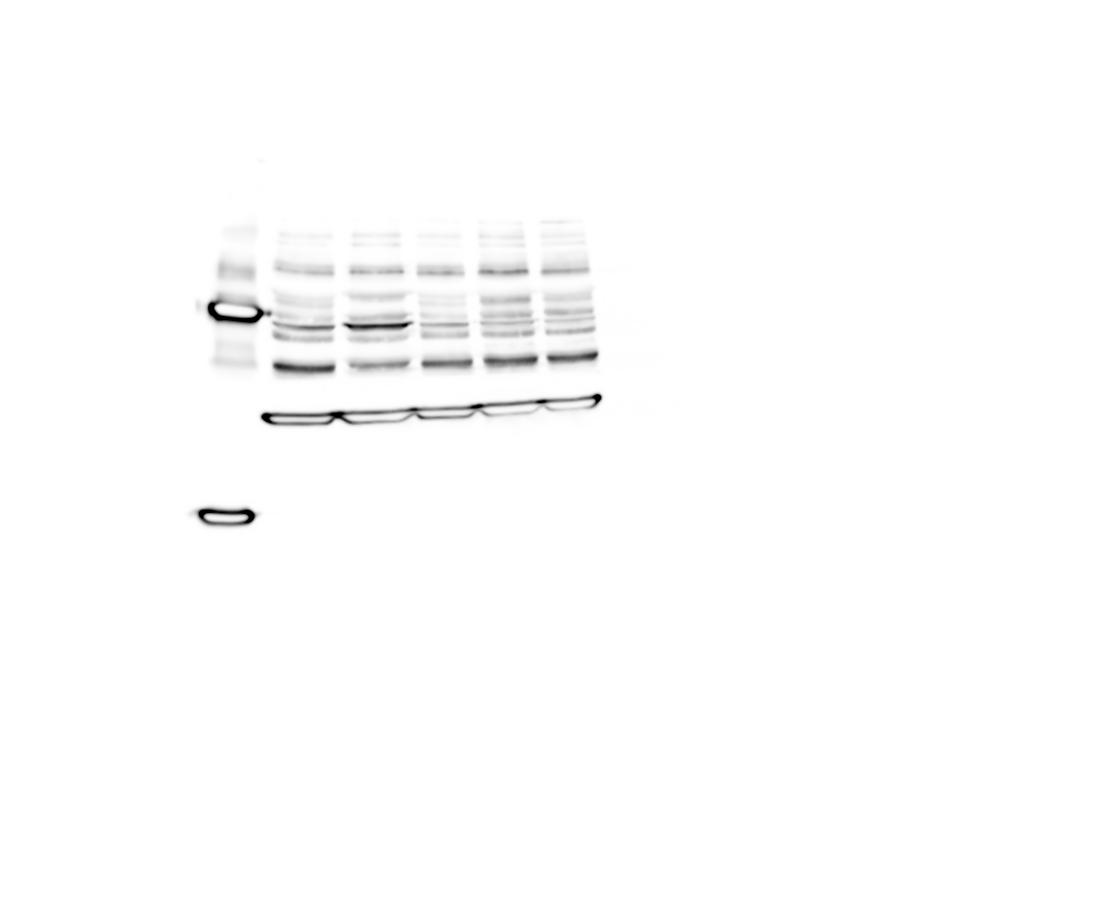

Supplement: Supplemental Information 2 [file peerj-13-19135-s002.zip › IMG-20240427-WA0034.jpg]

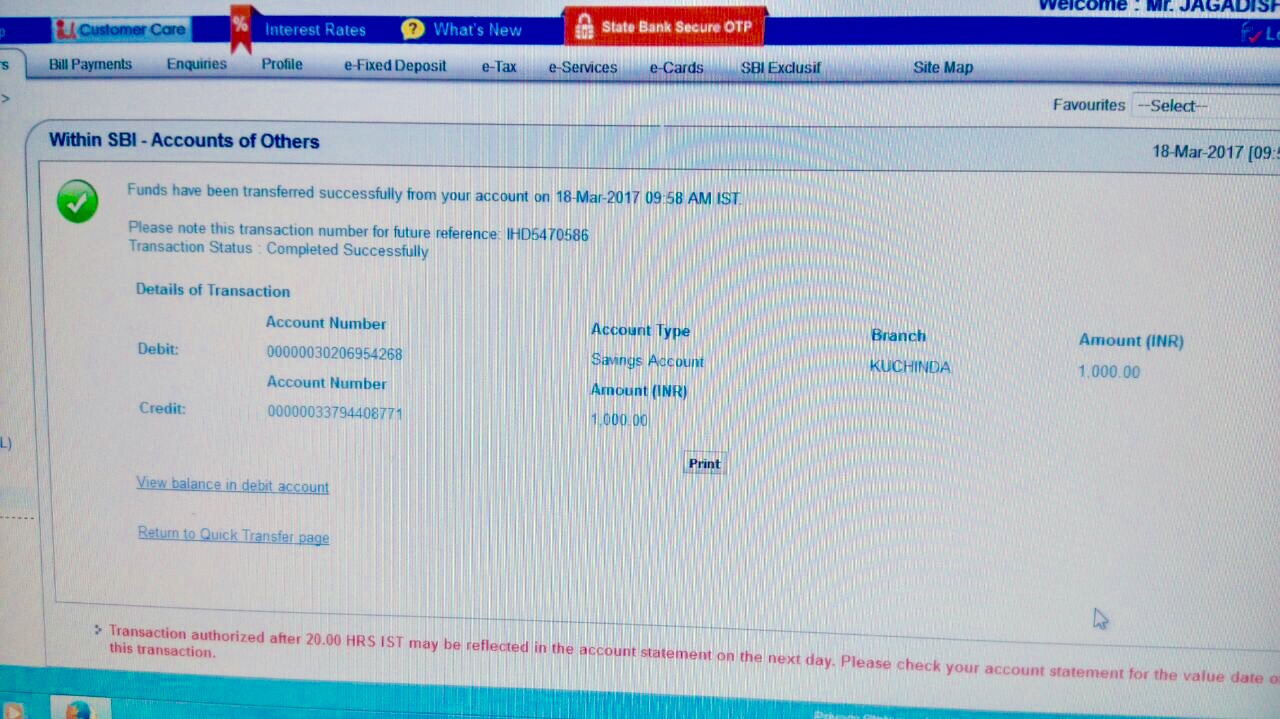

Supplement: Supplemental Information 2 [file peerj-13-19135-s002.zip › IMG-20240427-WA0035.jpg]

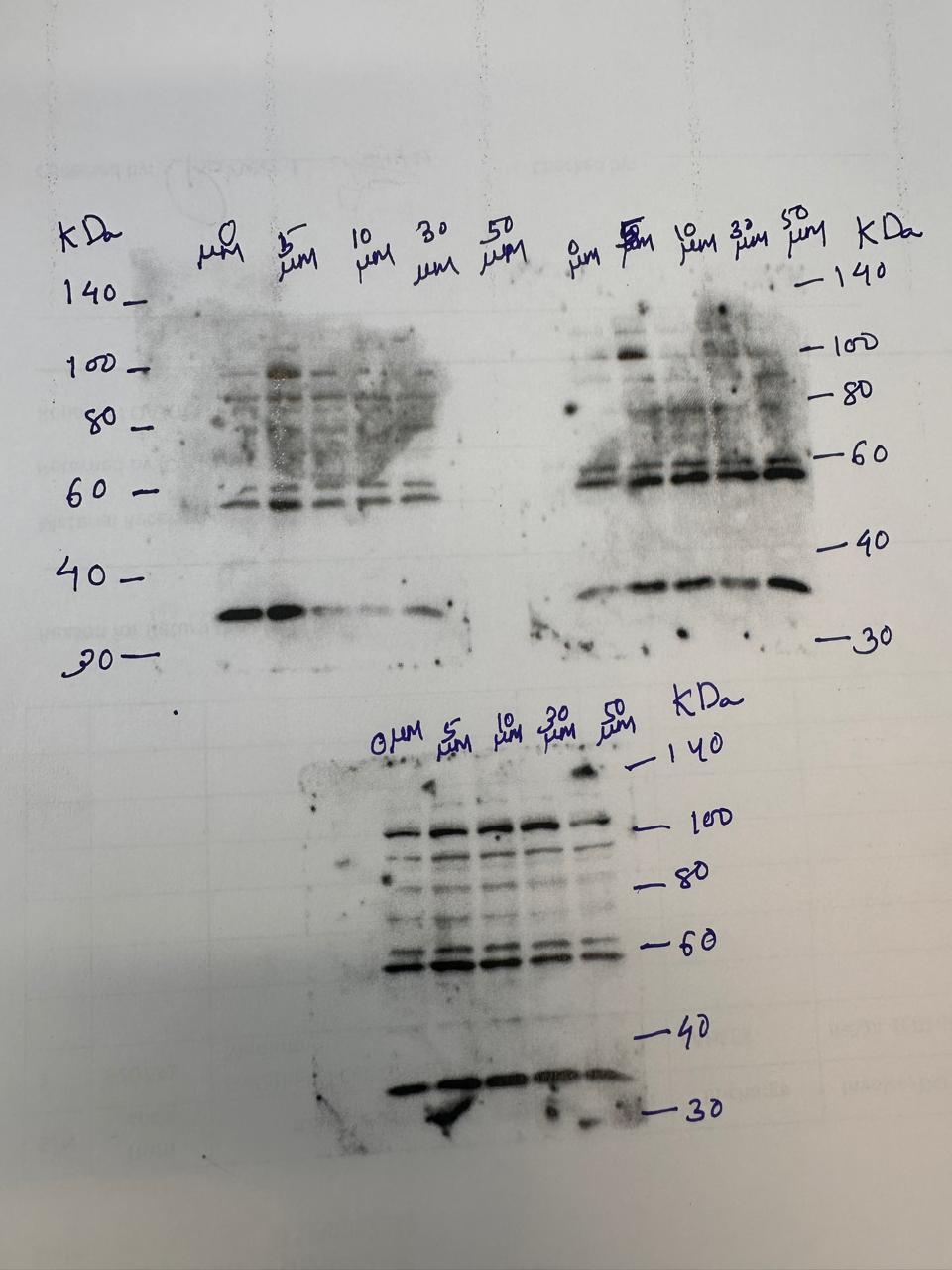

Supplement: Supplemental Information 2 [file peerj-13-19135-s002.zip › IMG-20240427-WA0036.jpg]

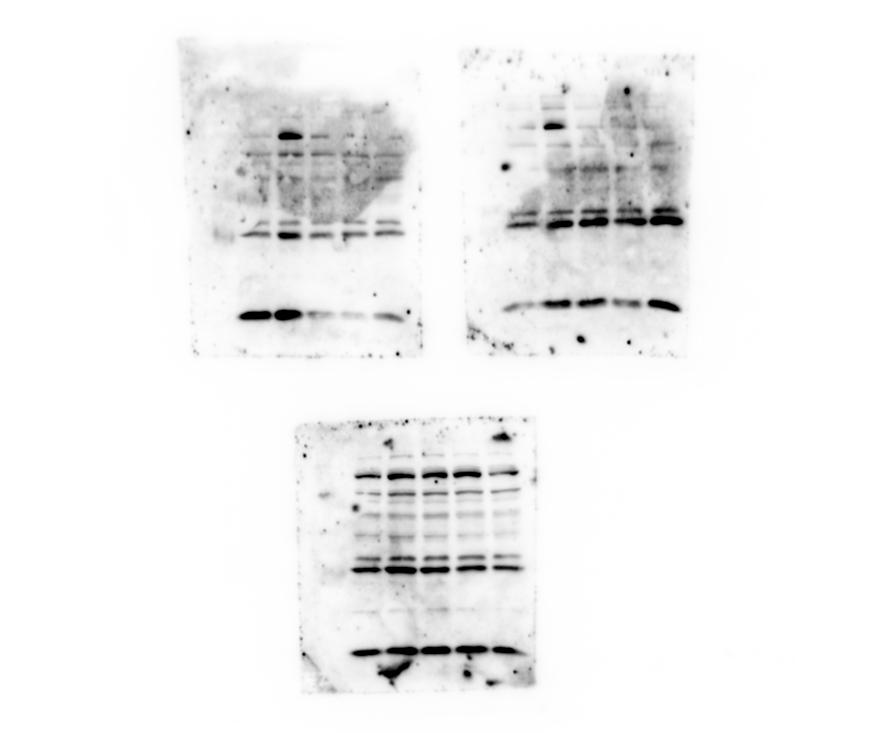

Supplement: Supplemental Information 2 [file peerj-13-19135-s002.zip › IMG-20240427-WA0037.jpg]
