# Supplementary material for: EGCG’s anticancer potential unveiled: triggering apoptosis in lung cancer cell lines through in vitro investigation
Source: PeerJ. 2025 Mar 26;13:e19135. doi: 10.7717/peerj.19135 (PMC11954466; doi:10.7717/peerj.19135)
Supplement: Supplemental Information 12 [file peerj-13-19135-s012.docx]

**Table 19. Pairwise Comparison of the A549 Concentrations (µM) pAKT between different concentration**

| Tukey Post-Hoc Test – A549 Concentrations (µM) pAKT | | | | | | | | | | | |
| --- | --- | --- | --- | --- | --- | --- | --- | --- | --- | --- | --- |
|  | |  | | **Control group (0 μM)** | | **Low-dose treatment group (5 μM)** | | **Middle dose group (30 μM)** | | **High-dose treatment group (50 μM)** | |
| Control group (0 μM) |  | Mean difference |  | — |  | 0.0540 | ** | 0.1380 | *** | 0.2220 | *** |
|  |  | p-value |  | — |  | 0.002 |  | < .001 |  | < .001 |  |
| Low-dose treatment group (5 μM) |  | Mean difference |  |  |  | — |  | 0.0840 | *** | 0.1680 | *** |
|  |  | p-value |  |  |  | — |  | < .001 |  | < .001 |  |
| Middle dose group (30 μM) |  | Mean difference |  |  |  |  |  | — |  | 0.0840 | *** |
|  |  | p-value |  |  |  |  |  | — |  | < .001 |  |
| High-dose treatment group (50 μM) |  | Mean difference |  |  |  |  |  |  |  | — |  |
|  |  | p-value |  |  |  |  |  |  |  | — |  |
| Note. * p < .05, ** p < .01, *** p < .001 | | | | | | | | | | | |
|  | | | | | | | | | | | |

 The Pairwise Comparison of the A549 Concentrations (µM) pAKT between different concentration depicted statistically significant difference between concentration (p<0.05)
