# Supplementary material for: EGCG’s anticancer potential unveiled: triggering apoptosis in lung cancer cell lines through in vitro investigation
Source: PeerJ. 2025 Mar 26;13:e19135. doi: 10.7717/peerj.19135 (PMC11954466; doi:10.7717/peerj.19135)
Supplement: Supplemental Information 13 [file peerj-13-19135-s013.docx]

**Table 20. Details of products, supply source and catalogue numbers**

| **Product** | **Supply Source** | **Catalogue Number** |
| --- | --- | --- |
|  |  |  |
| MTT | Thermofisher Scientific USA | M6494 |
| Roswell Park Memorial Institute 1640 media, | Himedia, India | AL162A |
| 1% penicillin-streptomycin, | Himedia, INdia | AO28 |
| 10% fetal bovine serum (FBS) | Sigma, USA | F7524 |
| PI3K | Abcam, USA | ab40755 |
| phosphorylated PI3K (p-PI3K) | Abcam, USA | ab235266 |
| AKT | Abcam, USA | ab81282 |
| phosphorylated AKT (p-AKT) | Abcam, USA | ab8932 |
